# Separation of Taurine and Sodium Sulfate from Simulated Mother Liquor by Electrodialysis and Process Optimization

**DOI:** 10.3390/membranes16080253

**Published:** 2026-07-23

**Authors:** Huiting Zhu, Douyan Cao, Jigang Zhao

**Affiliations:** School of Chemical Engineering, East China University of Science and Technology, Shanghai 200237, China; zhu_huiting829@163.com (H.Z.); y30230233@mail.ecust.edu.cn (D.C.)

**Keywords:** electrodialysis, taurine, sodium sulfate, desalination, process integration

## Abstract

To address the high energy demand and product losses associated with separating taurine from sodium sulfate (Na_2_SO_4_) in the ethylene oxide route to taurine, electrodialytic desalination of a simulated taurine/Na_2_SO_4_ mother liquor was investigated. The effects of applied voltage, circulation flow rate, and initial feed concentration on the desalination rate, product purity, taurine recovery, current efficiency, specific energy consumption, and membrane productivity were evaluated. Ion-transport behavior was further examined using COMSOL Multiphysics^®^ 6.3. At 14 V, a circulation flow rate of 200 L/h, and initial taurine and Na_2_SO_4_ concentrations of 100 and 68 g/L, respectively, the process achieved a taurine purity of 99.8% and a recovery of 98.9%. The specific electrical energy consumption of the electrodialysis unit was 0.56 kWh/kg Na_2_SO_4_, and the membrane productivity was 0.49 kg Na_2_SO_4_/(m^2^·h). One of the key findings of this work is that the low-salt stage plays a dominant role in process economics. This observation led to a simple endpoint-control strategy. The ED operation is stopped when the Na_2_SO_4_ concentration in the dilute compartment drops to about 2 g/L. This avoids prolonged operation under inefficient conditions and reduces ED energy consumption by 16.5%. Within the binary simulated system and the defined cost boundary, the proposed process provided a higher taurine recovery and a lower estimated separation cost than the conventional crystallization route. These results demonstrate the laboratory-scale feasibility of electrodialysis for desalting simulated taurine mother liquor.

## 1. Introduction

Taurine (2-aminoethanesulfonic acid) is a sulfur-containing amino acid with several physiological functions and is widely used in infant formula, functional beverages, pharmaceuticals, and feed additives [1,2,3]. With the growing demand for health-related products and fine chemicals, increasingly stringent requirements are being placed on the purity, recovery, and environmental performance of taurine production processes [4]. Taurine is currently produced mainly by the ethylene oxide route, which uses readily available raw materials and is well established at the industrial scale. However, this route generates a substantial amount of sodium sulfate (Na_2_SO_4_) as a by-product. Efficient separation of taurine and Na_2_SO_4_ with low energy consumption and minimal product loss is therefore essential for increasing product recovery, reducing process energy demand, and alleviating the wastewater-treatment burden.

Conventional taurine separation processes rely mainly on phase-change operations, including evaporation, concentration, cooling crystallization, and recrystallization. These processes separate taurine from Na_2_SO_4_ primarily on the basis of differences in solubility and its temperature dependence. However, as reported in studies on amino acid solubility and crystallization behavior, the presence of inorganic salts can significantly influence the solubility and nucleation kinetics of amino acids through salting-in/out effects and co-crystallization, which may compromise separation performance in practical operations [5,6]. In industrial practice, multiple crystallization or recrystallization steps are often required to ensure product purity, while mother liquor recycling is used to improve the overall recovery [7]. These measures increase the evaporation duty, energy consumption, and wastewater-treatment burden. Conventional crystallization therefore involves an inherent trade-off among product purity, recovery, and energy efficiency, which constrains the development of more sustainable and intensified taurine production processes.

Ion-exchange-based technologies have recently been investigated for separating taurine from Na_2_SO_4_ [8,9,10]. Electrodialysis (ED) is an electrically driven membrane process in which ions are selectively transported through ion-exchange membranes, and it has been applied to the desalination of organic acids, amino acids, and fermentation broths [11,12,13,14]. In a taurine/Na_2_SO_4_ solution, Na^+^ and SO_4_^2−^ migrate through cation-exchange membranes (CEMs) and anion-exchange membranes (AEMs), respectively, into the concentrate compartment. Near neutral pH, taurine has a degree of dissociation below 10^−5^ and exists predominantly as a zwitterion with a low net charge, which favors its retention in the dilute compartment. ED may therefore reduce the number of phase-change steps required in conventional crystallization, thereby lowering the separation energy and improving taurine recovery.

Early studies on ED separation of taurine from Na_2_SO_4_ demonstrated the technical feasibility of this approach. Konarev reported that introducing ED into taurine production markedly improved product quality, eliminated energy-intensive operations, and removed poisonous gas emissions [15]. In an early investigation, Zhao et al. achieved a taurine purity of 98.5% and a recovery of 78.1% from a taurine/Na_2_SO_4_ mixed solution using ED [16]. More recently, Eliseeva and Kharina comprehensively reviewed electromembrane desalination of amino acid solutions, examining the influence of various mineral cations (Na^+^, K^+^, NH_4_^+^) and anions (NO_3_^−^, SO_4_^2−^, Cl^−^) on ion transport and membrane current–voltage characteristics [17]. Studies on phenylalanine-salt systems have further revealed that electroconvection at overlimiting current modes can increase amino acid loss, and that membrane ion-exchange resin content significantly affects separation efficiency [18].

Studies on ED applied to the taurine/Na_2_SO_4_ system have been primarily limited to feasibility validation. The effects of operating parameters on mass-transfer behavior under high salt concentrations remain insufficiently understood. Endpoint regulation of the ED process coupled with crystallization for energy consumption control has not been reported, and the specific advantages of ED over crystallization have not been quantitatively evaluated. In this study, conventional ED was used to desalt a binary simulated taurine/Na_2_SO_4_ mother liquor. The effects of applied voltage, circulation flow rate, and initial feed concentration on the desalination rate, taurine purity and recovery, specific energy consumption, and membrane productivity were systematically evaluated. Ion-transport behavior was examined using a COMSOL Multiphysics^®^ model, and the effect of endpoint selection on process energy consumption was analyzed. We hypothesize that the deterioration of ED performance is mainly governed by concentration-dependent transport limitation rather than membrane selectivity loss. Therefore, identifying the critical salt concentration and establishing an endpoint-control strategy may provide a more energy-efficient route. The results provide experimental data for evaluating ED as a potential separation step in the production of taurine by the ethylene oxide route.

## 2. Materials and Methods

### 2.1. Materials and Equipment

Taurine (TAU, analytical grade) was supplied by Luofu Pharmaceutical Technology Co., Ltd., Jiaxing, China. Anhydrous sodium sulfate (Na_2_SO_4_, purity ≥ 99.9%) was obtained from Shanghai Titan Scientific Co., Ltd., Shanghai, China. Deionized water was produced in-house. Formaldehyde, phenolphthalein, and a standardized NaOH solution (0.1 mol/L) were purchased from Shanghai Macklin Biochemical Technology Co., Ltd., Shanghai, China.

An SED-30 laboratory-scale electrodialysis stack (Shanghai Sanji New Material Co., Ltd., Shanghai, China) was used. The stack was equipped with polyethylene (PE)-based anion-exchange membranes functionalized with quaternary ammonium groups and PE-based cation-exchange membranes functionalized with sulfonic acid groups. The spacer used in the stack is of a square-mesh support type with a grid size of 1 cm × 1 cm and a filament width of 1 mm. The electrode material is Ti/Pt (titanium-coated platinum). The principal equipment specifications are listed in Table 1.

### 2.2. Electrodialysis Setup and Procedure

**Figure 1:**
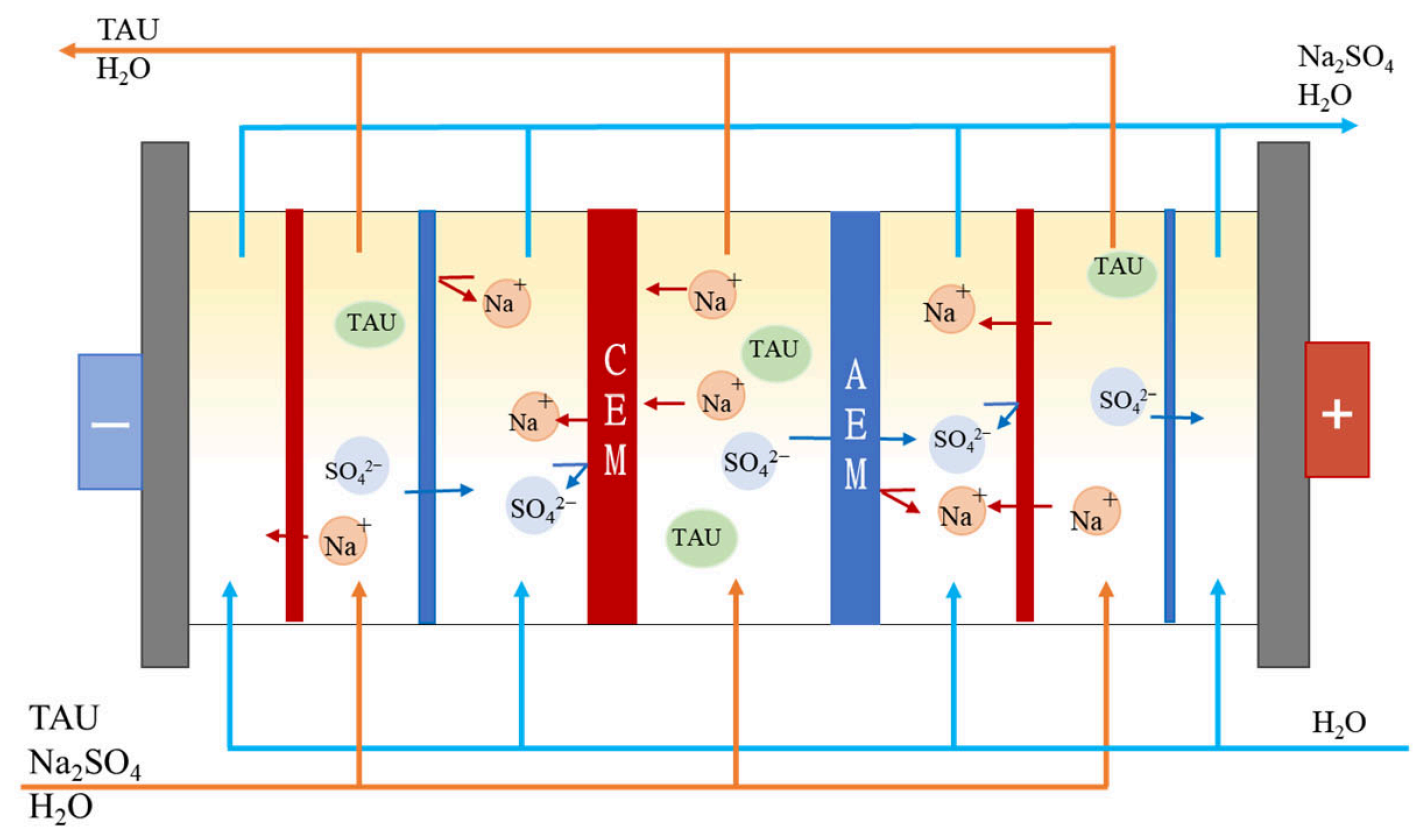
Schematic of the electrodialysis process for separating taurine and Na_2_SO_4_. The orange arrows indicate the flow in the dilute compartment, and the blue arrows indicate the flow in the concentrate compartment.

The experiments were conducted in batch ED mode. The dilute reservoir was filled with 1 L of a mixed solution containing taurine and Na_2_SO_4_ at a fixed mass ratio of 1:0.68 to simulate the industrial mother liquor from the ethylene oxide route. The concentrate reservoir was initially filled with 1 L of deionized water. The interconnected electrode loop serving both the anode and cathode was filled with 1 L of a 3 wt.% Na_2_SO_4_ solution to limit pH changes caused by electrode reactions. Deionized water was used in the concentrate compartment at the beginning of each batch experiment. Although this practice inevitably increased the initial electrical resistance of the stack and may have temporarily reduced the current efficiency, it was adopted to establish a well-defined initial state with zero background salt concentration. This allowed the salt flux across the membranes to be quantified directly from conductivity changes in the concentrate stream. Figure 1 schematically shows the separation principle of taurine and Na_2_SO_4_ by electrodialysis.

The circulation flow rates in the dilute and concentrate loops were set to the same value ((50–200 L/h) as specified for each experiment), whereas the electrode-loop flow rate was fixed at 100 L/h. After the flow rates had stabilized, the initial conductivity, temperature, and pH were recorded. The DC power supply was then switched on at the prescribed voltage (8–16 V). During operation, Na^+^ and SO_4_^2−^ through the CEM and AEM, respectively, into the concentrate compartment, while taurine was largely retained in the dilute compartment. Conductivity, pH, temperature, stack voltage, and current were recorded every 5 min. Each run was terminated when the current decreased to 0.1 A. At the end of the run, 100 g samples were collected from both the dilute and concentrate reservoirs, dried at 80 °C for 4 h and subsequently at 60 °C for 12 h, and sealed for analysis. Each experiment was performed in triplicate. Desalination-rate data are reported as medians, whereas recovery, purity, energy consumption, and membrane productivity are reported as mean values. The desalination rate is reported as the median value of triplicate runs because the conductivity readings exhibited relatively large fluctuations toward the end of the experiments, particularly when the salt concentration became low, and the use of the mean would have introduced bias due to these endpoint deviations. The median, being less sensitive to extreme fluctuations, provides a more representative description of the desalination trend.

### 2.3. Limit Current Density Measurement

**Figure 2:**
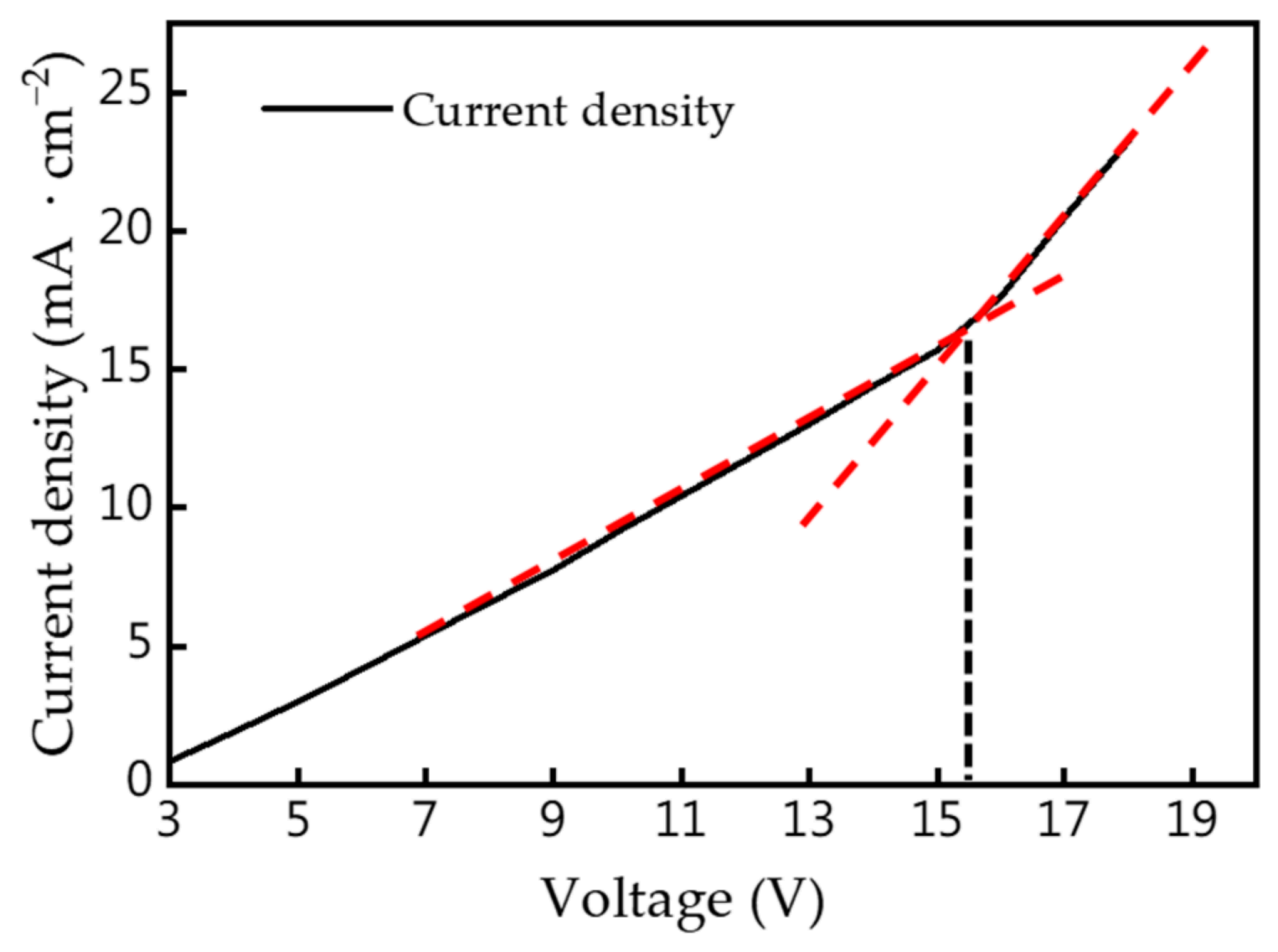
Determination of the limiting current density from the current–voltage curve. The red and black dashed lines are auxiliary lines indicating the intersection point where the slope of the current–voltage curve changes.

The limiting current density (i_lim_) was determined using the current–voltage curve method [19]. A 2 L solution containing 68 g/L Na_2_SO_4_ (100 g/L taurine) corresponding to the salt concentration in the simulated mother liquor, was prepared. The dilute and concentrate reservoirs were each filled with 1 L of this solution, and the outlets were cross-connected to maintain approximately constant concentrations during the measurement. The electrode loop was filled with 1 L of 3 wt.% Na_2_SO_4_ solution. The applied voltage was increased stepwise from 3 V to higher values in increments of 1 V and the steady-state current was recorded at each voltage. As shown in Figure 2, the intersection associated with the marked change in slope of the current–voltage curve corresponded to an approximate limiting current density of 16.24 mA/cm^2^ at 15.4 V.

The i_lim_ measured under constant-concentration conditions provides a practical reference for selecting the applied voltage, rather than a fixed threshold that applies uniformly throughout the entire batch desalination process. In actual operation, the dilute compartment concentration decreases continuously, and the i_lim_ varies accordingly. Therefore, a current density that exceeds the measured i_lim_ under steady-state conditions may not necessarily cause concentration polarization when the bulk concentration is high, while a current density below the measured i_lim_ may still cause polarization when the salt concentration becomes low. The actual current density evolution during each run is therefore the more relevant indicator for assessing polarization.

Above approximately 15 V, the current–voltage curve exhibits a second slope increase, which deviates from the classical plateau behavior expected for simple salt solutions. At overlimiting current densities, water dissociation at the membrane surface generates H^+^ and OH^−^ ions, which interact with taurine molecules through protonation/deprotonation, increasing the number of charged species available for ion transport [20]. This barrier effect allows the current to rise further despite mass-transfer limitations.

### 2.4. COMSOL Simulation

#### 2.4.1. Model Setup and Parameters

A two-dimensional mass-transfer model of the ED cell was developed using the Electrodeposition Module in COMSOL Multiphysics^®^. Ion transport was described by the Nernst–Planck equation (Equation (1)), and ionic mobility (*u_m,i_*) was calculated from the Nernst–Einstein relationship (Equation (2)) [21]:

(1) $$J_{i}\;=\;-D_{i}\;(\frac{{\mathrm{dC}}_{i}}{\mathrm{dx}}\;+\;z_{i}C_{i}\frac{F}{\mathrm{RT}}\frac{d\varphi }{\mathrm{dx}}\;+\;C_{i}\frac{\mathrm{dln}\gamma_{i}}{\mathrm{dx}})$$

(2) $$u_{m,i}=\frac{D_{i}}{RT}$$

where *J_i_* (mol/(m^2^·s)) is the total flux of ion i, *D_i_* (m^2^/s) is the diffusion coefficient, *γ_i_* is the activity coefficient, *C_i_* (mol/m^3^) the concentration, *z_i_* is the charge number, *F* the Faraday constant (96,485 C/mol), *R* is the gas constant [8.314 J/(mol∙K)], *T* the temperature (K), and *dφ*/*dx* is the electric-potential gradient. In the present model, the activity-coefficient term in Equation (1) was retained only for completeness of the theoretical framework. For practical implementation, ideal solution behavior was assumed (*γ_i_* = 1) and the activity-coefficient gradient was neglected. This simplification is acceptable for qualitative trend analysis, which is the primary purpose of the simulation in this study.

**Figure 3:**
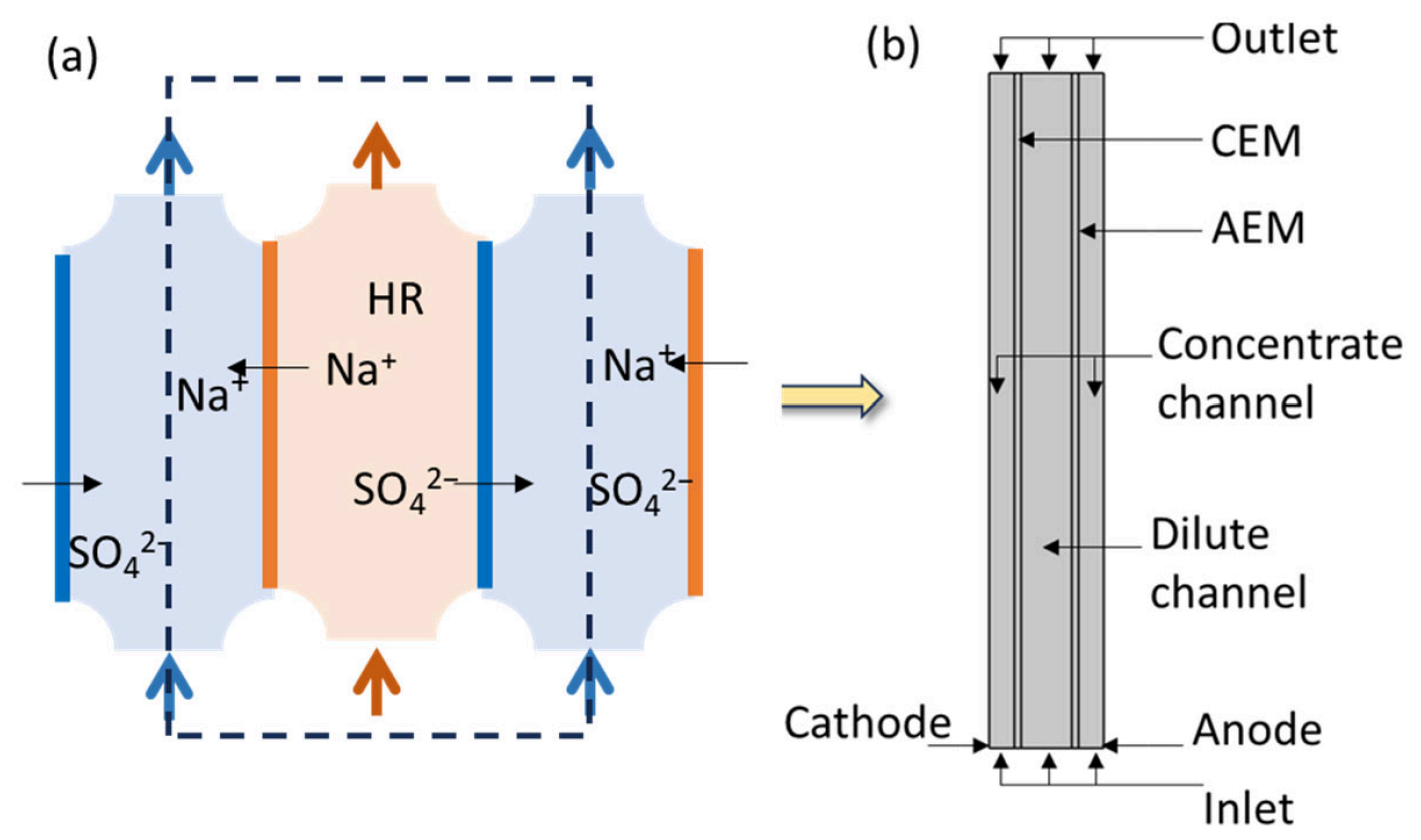
(**a**) Schematic of one electrodialysis unit; (**b**) two-dimensional COMSOL model. Orange arrows indicate the flow in the dilute compartment; blue arrows indicate the flow in the concentrate compartment; black arrows indicate the direction of ion migration.

Figure 3a shows a schematic two-dimensional ED unit. The region enclosed by the dashed rectangle was selected as the computational domain, as illustrated in Figure 3b. Donnan equilibrium was imposed at the membrane/solution interfaces, and charge conservation was represented using the water-based electroneutrality condition. The computational domain of the two-dimensional model is shown in Figure 3b, covering one ED unit cell (one dilute channel flanked by cation- and anion-exchange membranes and adjacent concentrate channels) [22]. The boundary layers adjacent to the membrane surfaces are not pre-defined but emerge from the simulation as regions of steep concentration gradients, reflecting the onset of concentration polarization. The positive flux direction is defined as from the dilute to the concentrate side (left to right in Figure 3b).

Taurine transport is not explicitly simulated because taurine is predominantly zwitterionic and electrically neutral under the experimental pH conditions, and its retention is confirmed by the high purity and recovery values obtained from titration. The principal model parameters are listed in Table 2, including the ionic diffusion coefficients [23], membrane thickness, channel width, and operating variables corresponding to the experimental ranges.

#### 2.4.2. Mesh Independence

**Figure 4:**
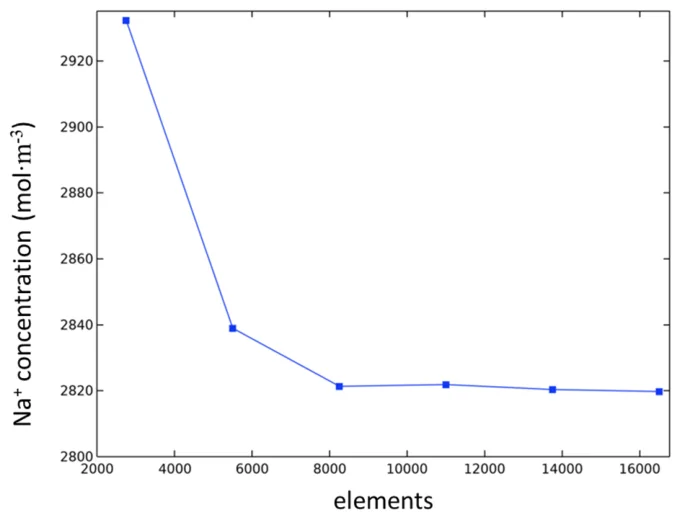
Mesh-independence test.

Mesh independence test was evaluated by monitoring the average Na^+^ concentration at the CEM surface on the cathode side. As shown in Figure 4, the calculated concentration became effectively independent of the mesh when the number of elements exceeded 8250. Accordingly, a mesh containing 8250 elements was used in subsequent simulations to balance numerical accuracy and computational cost.

The COMSOL model presented here was developed as a qualitative interpretation tool to visualize the ion-transport behavior predicted by the Nernst–Planck equations under different operating conditions. The model has not been quantitatively validated against experimental data, and its outputs are intended to illustrate the trends observed in the experiments rather than to provide accurate quantitative predictions. Quantitative validation would require detailed inputs such as membrane transport properties, boundary-layer thickness, and concentration-dependent diffusion coefficients which are beyond the scope of the present study.

### 2.5. Data Analysis

To quantify the Na_2_SO_4_ concentration, a calibration curve was first established. A series of Na_2_SO_4_ standard solutions was prepared, and the Na^+^ concentration of each solution was accurately determined by ICP-MS. The corresponding Na_2_SO_4_ concentration was then calculated from the Na^+^ content. The conductivity of each standard solution was also measured using a calibrated conductivity meter. A regression equation was fitted between the measured conductivity and the Na_2_SO_4_ concentration, as shown in Equation (3) (R^2^ = 0.99):

(3) $$C\;=\;176.19-\frac{176.06}{1+{\left(\sigma /182.19\right)}^{0.93}}$$

where *C* (g/L) is the Na_2_SO_4_ concentration and *σ* (mS/cm) the conductivity. In subsequent ED experiments, the Na_2_SO_4_ concentration was determined from the measured conductivity using this calibration curve, without repeating ICP-MS analysis for each sample.

Taurine was quantified by titration with standardized NaOH solution in the presence of formaldehyde. Taurine is a zwitterionic amino acid and cannot be directly titrated with a strong base. When formaldehyde is added, it reacts with the amino group (-NH_2_) of taurine to form N-methylol derivatives, which eliminates the basicity of the amino group and releases protons. This allows the sulfonic acid group (-SO_3_H) to be titrated with standardized NaOH solution [24]. The endpoint was determined by a color change in phenolphthalein. Taurine purity (*W*, %) and recovery (*X*, %) were calculated by:

(4) $$W=\frac{\left(\Delta V_{1}-\Delta V_{0}\right)C_{\mathrm{NaOH}}\times M}{m_{0}}100\%$$

(5) $$X=1-\frac{m_{2}\times W_{1}}{m_{1}}\times 100\%$$

where Δ*V*_1_ and Δ*V*_0_ (L) are the volumes of NaOH solution consumed for the sample and the blank respectively; *C_NaOH_* is the NaOH concentration (0.1 mol/L); *M* is the molar mass of taurine (125.147 g/mol); *m*_0_ (g) is the sample mass (0.2 g); *m*_1_ (g) is the initial mass of taurine in the dilute compartment; *m*_2_ (g) is the total solute mass recovered from the concentrate compartment; and *W*_1_ (%) is the taurine mass fraction in the concentrate compartment determined by titration. The recovery (X, %) is calculated as the fraction of taurine retained in the dilute compartment.

The method was validated under the experimental conditions. The average recovery was 100.2% (RSD = 0.42%, n = 3), and the RSD for repeated measurements was below 0.5%. These results confirm that the titration method is reliable and suitable for taurine quantification in this study.

Current efficiency (*CE*, %), specific energy consumption for Na_2_SO_4_ removal (*EC*, kJ/kg Na_2_SO_4_) and membrane productivity [*MP*, kg Na_2_SO_4_/(h·m^2^)] were calculated by [25]:

(6) $$\mathrm{CE}\;=∆n\cdot e\cdot F/N\int_{0}^{\;t\;}\;I\;\mathrm{dt}$$

(7) $$EC=\int_{0}^{\;t}\;U\cdot I\;\mathrm{dt}/m$$

(8) MP=3600·m/(t·A·N)

where *n* (mol) is the amount of transferred Na^+^ or SO_4_^2−^, *e* is the charge number, *N* is the number of cell pairs (10), *I* (A) is the current, *U* (V) is the stack voltage, *t* (s) is the operating time, *A* (m^2^) is the effective membrane area of one membrane sheet, and *m* (kg) is the mass of Na_2_SO_4_ mass in the concentrate compartment at the end of the run. In this case, the average Na_2_SO_4_ flux was used to represent membrane productivity.

The estimated separation cost (USD/kg taurine) included electricity, steam (for subsequent concentration/crystallization), and membrane replacement. The membrane price was 220 USD/m^2^ based on a supplier quotation, and the assumed membrane service life was 5 years. The membrane-replacement cost (*MC*, USD/kg taurine) was calculated as follows:

*MC* = (220 × 2 × 0.68)/(5 × 8000 × *MP*) (9)

where *MP* is the membrane productivity [kg Na_2_SO_4_/(m^2^·h)], and “8000” is the assumed annual operating time (h), “0.68” represents the mass ratio of Na_2_SO_4_ to taurine in the feed (taurine: Na_2_SO_4_ = 1:0.68), The factor “×2” accounts for the fact that each cell pair in the electrodialysis stack consists of two membranes: one cation-exchange membrane and one anion-exchange membrane. Electricity and steam prices were taken as 0.1 USD/kWh and 37 USD/t, respectively, based on representative industrial prices in first-tier Chinese cities.

## 3. Results and Discussion

### 3.1. Effect of Applied Voltage

At a baseline circulation flow rate of 150 L/h, the feed to the dilute compartment contained 68 g/L Na_2_SO_4_ and 100 g/L of taurine. The applied voltage was varied from 8 to 16 V (8, 10, 12, 14, and 16 V) to evaluate its effect on the separation performance.

**Figure 5:**
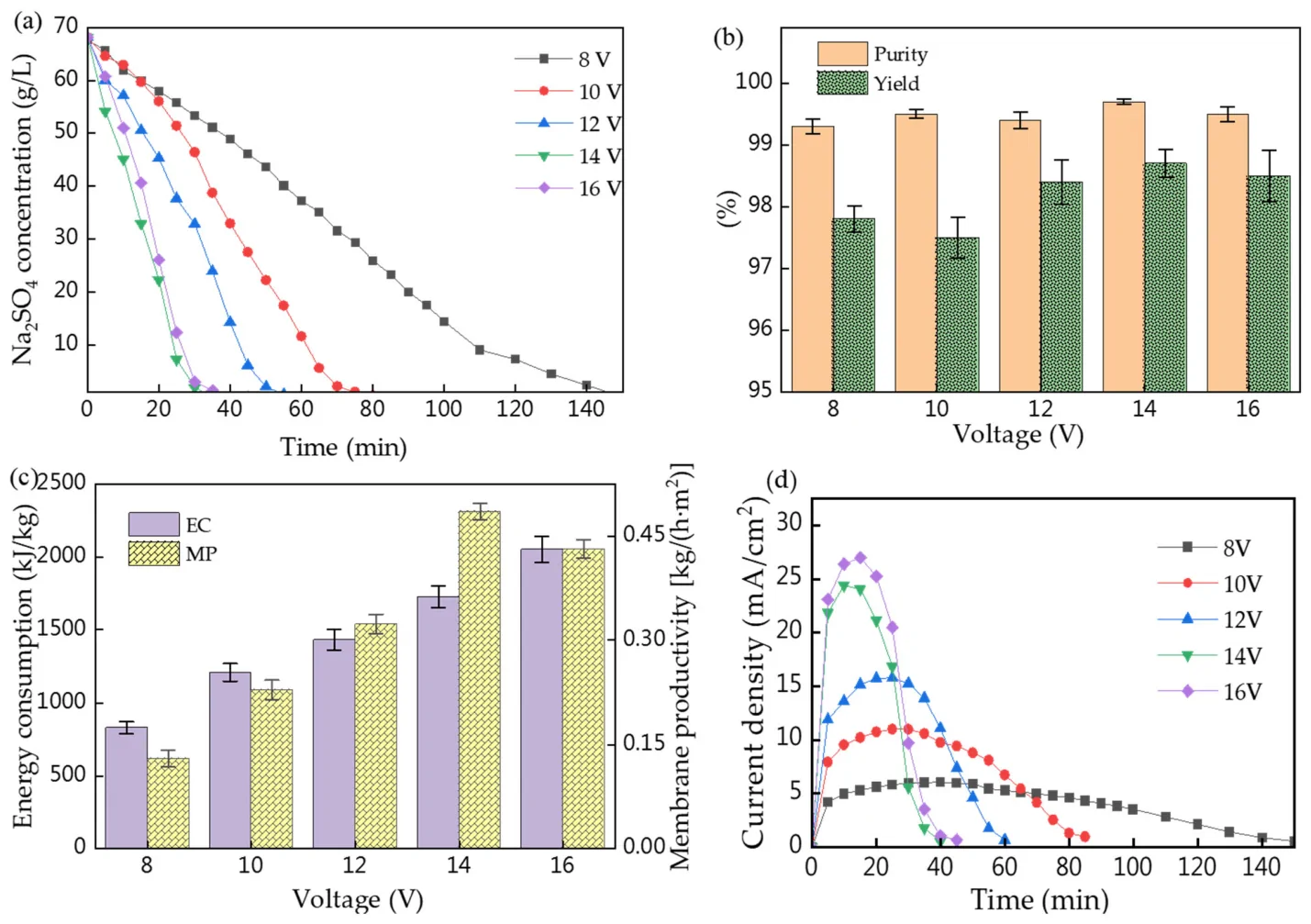
Effects of applied voltage on (**a**) the desalination profile, (**b**) taurine purity and recovery, and (**c**) specific energy consumption and membrane productivity, (**d**) current density.

**Figure 6:**
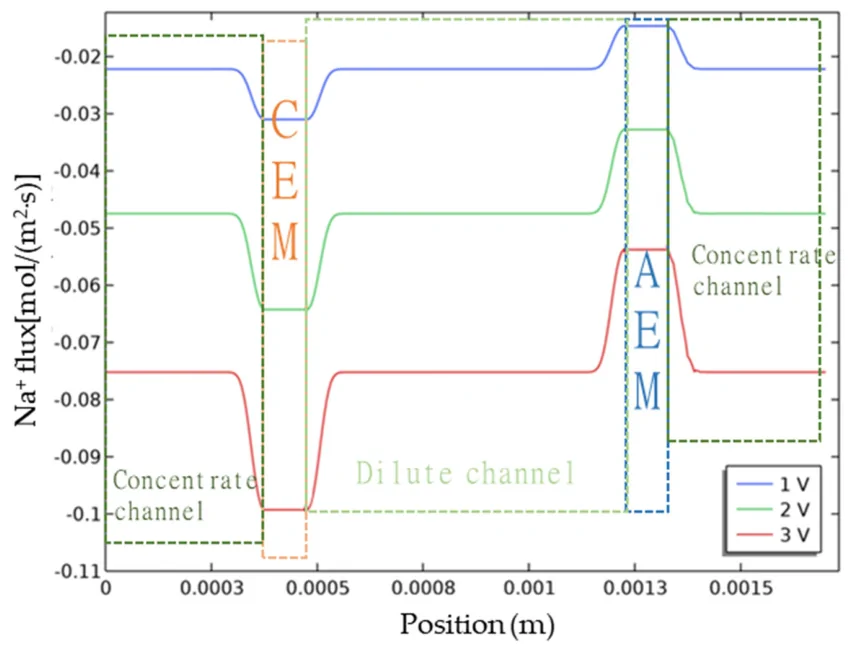
Simulated effect of applied voltage on the Na^+^ flux.

As shown in Figure 5a, the desalination rate increased as the applied voltage increased from 8 to 14 V. The simulated Na^+^ flux profiles in Figure 6 are presented as qualitative illustrations of the effect of applied voltage on the ion transport. The nearly flat regions at 0.0004 and 0.0013 m correspond to the ion-exchange membranes, whereas the regions with changing slopes on either side represent the membrane/solution boundary layers. While the simulations show a clear increase in Na^+^ flux with increasing unit voltage (from 1 to 3 V), consistent with the experimental desalination trends, the absolute flux values should not be interpreted as validated predictions due to the simplified nature of the model and the lack of quantitative calibration against the present experimental system.

When the voltage was further increased to 16 V, the desalination rate decreased. This behavior cannot be explained fully by the simplified ion-migration model and was therefore interpreted in conjunction with current density. To further examine the role of current density, the current density during each desalination run was recorded and plotted against time (Figure 5d). The peak current density increased with increasing applied voltage, which is expected from Ohm’s law. At 8–12 V, the current density curves remained relatively stable throughout the run, indicating that ion transport proceeded without significant mass-transfer limitation. At 14 V and 16 V, however, the curves became much steeper, reflecting a more rapid decline in current density as desalination progressed. The super-linear increase in current at higher voltages is characteristic of the overlimiting current regime in ion-exchange membrane systems. Water dissociation and electroconvection at the membrane/solution interface generate additional charge carriers and enhance mass transfer, respectively, leading to a current rise that exceeds the linear Ohm’s law prediction [20].

Notably, although 16 V gave the highest peak current density among all tested voltages, it did not result in a higher desalination rate than 14 V (Figure 5a). This apparent contradiction suggests that the additional current at 16 V did not contribute effectively to salt removal; instead, a considerable fraction of the applied current was consumed by side reactions such as water dissociation at the membrane/solution interfaces. This behavior is a typical manifestation of concentration polarization: once the operation exceeds the limiting current density, further voltage increase enhances parasitic processes rather than ion migration, leading to a decrease in current efficiency and an overall loss of desalination performance. In contrast, at 14 V, the balance between driving force and mass transfer was more favorable, resulting in the highest effective desalination rate among the conditions tested [26,27].

**Figure 7:**
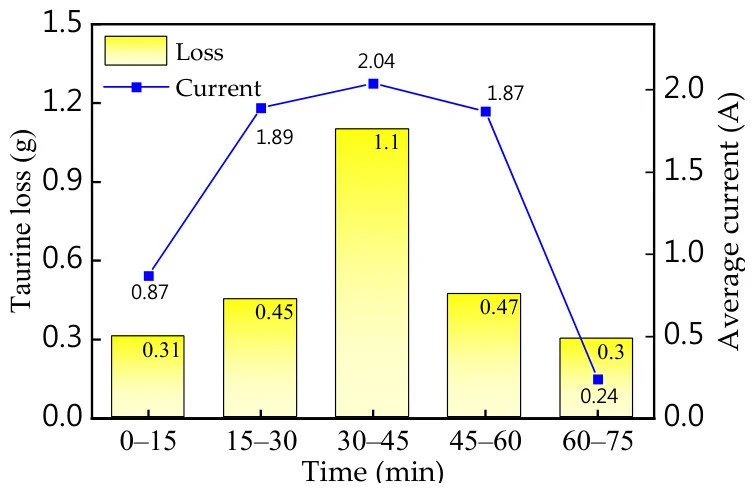
Relationship between taurine loss, operating time, and current (data obtained at 10 V).

Within the investigated voltage range, 14 V therefore provided a comparatively high desalination rate. As shown in Figure 5b, the differences in product purity among the voltage conditions were less than 0.2 percentage points, and all purities exceeded 99.5%. The highest measured purity was 99.7% at 14 V. The small variation among the voltage conditions may be attributable to slight differences in the endpoint reached in each run. The taurine recovery at 14 V was 98.7%, and no monotonic relationship between recovery and voltage was observed. Figure 7 indicates that taurine loss was affected by both operating time and current. The data in Figure 7 were collected from the run at 10 V, which was selected as an intermediate voltage level among the tested range. A higher voltage shortened the separation time and may therefore have reduced passive diffusive loss of taurine [28]; however, it also increased the current and may have promoted taurine transport associated with ion migration. These competing effects are consistent with the observed variation in taurine recovery.

Figure 5c shows that the applied voltage affected both the specific energy consumption and membrane productivity. The energy consumption per unit mass of Na_2_SO_4_ removed increased with voltage. This trend is likely associated with Joule heating: higher currents generate more resistive heat in the membranes and solution, thereby reducing current efficiency. The solution temperature increased slightly with increasing applied voltage (Table 3). The observed temperature rise ranged from 0.3 °C (at 8 V) to 1.4 °C (at 16 V), consistent with the expected Joule heating effect. However, given the small magnitude of these changes and the fact that all temperatures remained well below the membrane manufacturer’s recommended limit (40 °C), the thermal effect is unlikely to have significantly influenced membrane performance or separation efficiency under the tested conditions [29].

A paired *t*-test between the 8 V and 16 V groups gave *p* > 0.05, indicating that the temperature differences observed across the voltage range were not statistically significant at the 95% confidence level. This suggests that the thermal effect was minor under the present operating conditions. Therefore, the decline in current efficiency can also be attributed to other factors. Although the current increased with applied voltage, the current efficiency decreased slightly at higher voltages because a larger fraction of the applied current may be consumed by water dissociation and counter-ion transport rather than by Na_2_SO_4_ removal. Second, the higher current density at elevated voltages may have promoted concentration polarization near the membrane surfaces, increasing the membrane resistance and thus the energy required per unit of salt removed. Joule heating was a minor side-effect rather than a primary contributor to the observed increase in energy consumption. The dominant factor governing the voltage-dependence of energy consumption was therefore the trade-off between increased driving force and reduced current efficiency. In electrodialysis, temperature influences both membrane conductivity and ion mobility [30]. Higher temperatures generally increase ionic diffusivity and reduce solution viscosity, which can enhance ion transport across the membrane and improve current efficiency. However, as shown in Table 3, the solution temperature in all experiments remained below 40 °C, which is within the safe operating range specified by the membrane manufacturer. A portion of the additional energy at higher voltages may also be dissipated through electrode overpotential and ohmic losses at the electrode/solution interface [31], as well as through water dissociation associated with concentration polarization. The dominant factor governing the voltage-dependence of energy consumption was therefore the trade-off between increased driving force and reduced current efficiency.

Increasing the applied voltage also shortened the separation time substantially, thereby reducing the membrane area required to achieve a specified production capacity. Within an appropriate voltage range, increasing the voltage can therefore reduce the membrane cost per unit mass of product.

For an illustrative annual taurine production capacity of 500 t and an annual operating time of 8000 h, the production rate would be 62.5 kg taurine/h, and the Na_2_SO_4_ removal rate would be 42.5 kg/h. The estimated ED separation costs under these conditions are summarized in Table 4. Based on the separation performance and the estimated electricity and membrane replacement costs, 14 V gave the lowest combined cost among the tested voltages within the defined accounting boundary. This cost estimation considers only electricity and membrane replacement, and does not include other capital or operating costs.

### 3.2. Effect of Circulation Flow Rate

**Figure 8:**
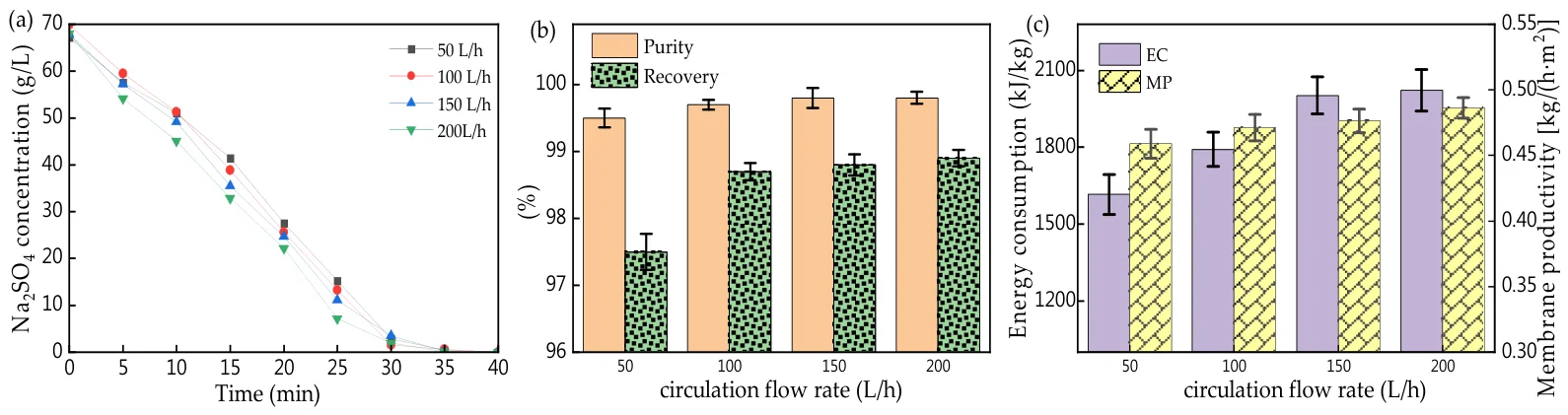
Effects of circulation flow rate on (**a**) the desalination profile, (**b**) taurine purity and recovery, and (**c**) specific energy consumption and membrane productivity.

**Figure 9:**
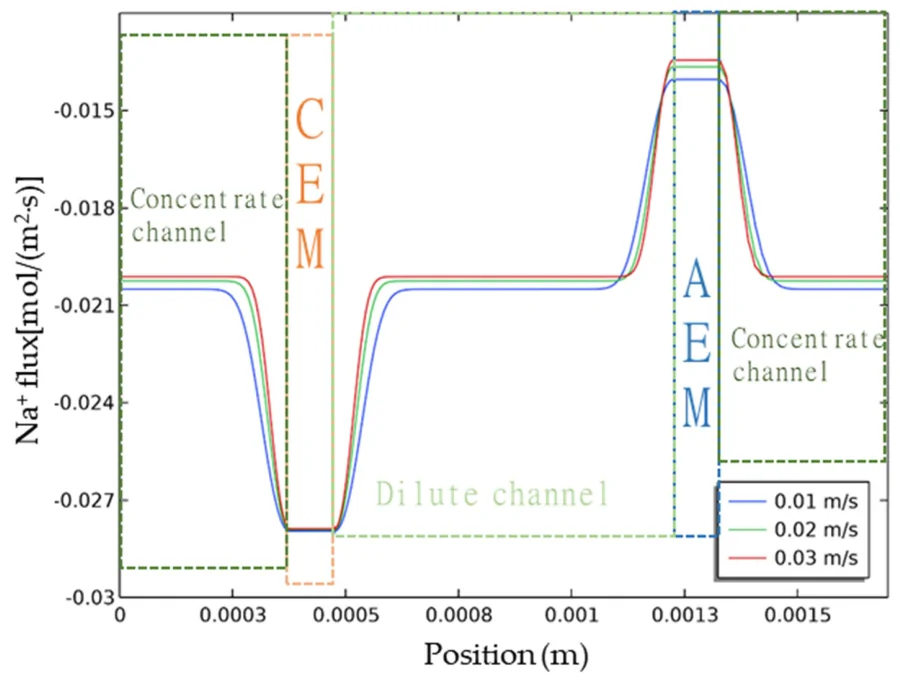
Simulated effect of circulation velocity on the Na^+^ flux.

At an applied voltage of 14 V and an initial Na_2_SO_4_ concentration of 68 g/L in the dilute feed, the circulation flow rate was varied among 50, 100, 150, and 200 L/h (corresponding to linear velocities of 1.5, 3.1, 4.6, and 6.2 cm/s, respectively). The effects of circulation flow rate on the desalination profile, product quality and economic indicators are shown in Figure 8. Increasing the flow rate had only a minor effect on the overall desalination rate, although a slight increase was observed during the initial stage, when the concentrate compartment contained deionized water. The simulation results in Figure 9 indicate that flow velocity had a limited effect on the ion flux but reduced the boundary-layer thickness, consistent with the experimental trends. According to the Nernst–Planck framework, a higher flow velocity decreases the diffusion-boundary-layer thickness and accelerates ion transport from the bulk solution to the membrane surface [32]. The taurine purity consequently increased slightly with flow rate (Figure 8b). The taurine recovery at 50 L/h was lower than those obtained at the higher flow rates. This result may be associated with weaker hydrodynamic mixing and a longer residence time of taurine near the membrane interface, which could increase its probability of crossing the membrane.

As shown in Figure 8c, increasing the circulation flow rate slightly increased the electrical energy consumption. Nevertheless, 200 L/h provided the highest taurine purity (99.8%) and recovery (98.9%) together with a comparatively high membrane productivity, and it was therefore selected for subsequent experiments. At this flow rate, the membrane-surface linear velocity was 6.2 cm/s. For process scale-up, the membrane-surface linear velocity, rather than the volumetric flow rate alone, should be used as the principal hydrodynamic scaling parameter. The effect of flow rate on separation performance was weaker than that of applied voltage; however, excessively low flow rates should be avoided because they may increase mass-transfer limitations within the stack.

### 3.3. Effect of Initial Feed Concentration

At a circulation flow rate of 150 L/h and an applied voltage of 14 V, the initial Na_2_SO_4_ concentration in the feed was varied among 34, 42.5, 51, 59.5, and 68 g/L. The taurine/Na_2_SO_4_ mass ratio was maintained at 1:0.68 in all experiments. The concentration-dependence experiments were conducted at a circulation flow rate of 150 L/h, which was the same condition used in the voltage optimization experiments to maintain a consistent hydrodynamic baseline.

**Figure 10:**
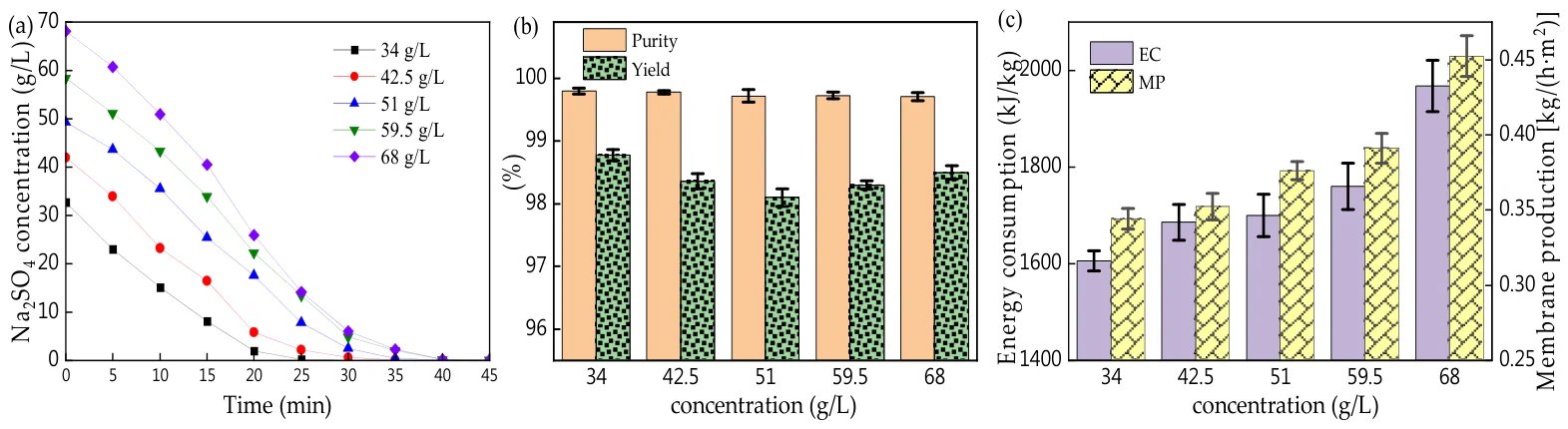
Effects of initial feed concentration on (**a**) the desalination profile, (**b**) taurine purity and recovery, and (**c**) specific energy consumption and membrane productivity.

**Figure 11:**
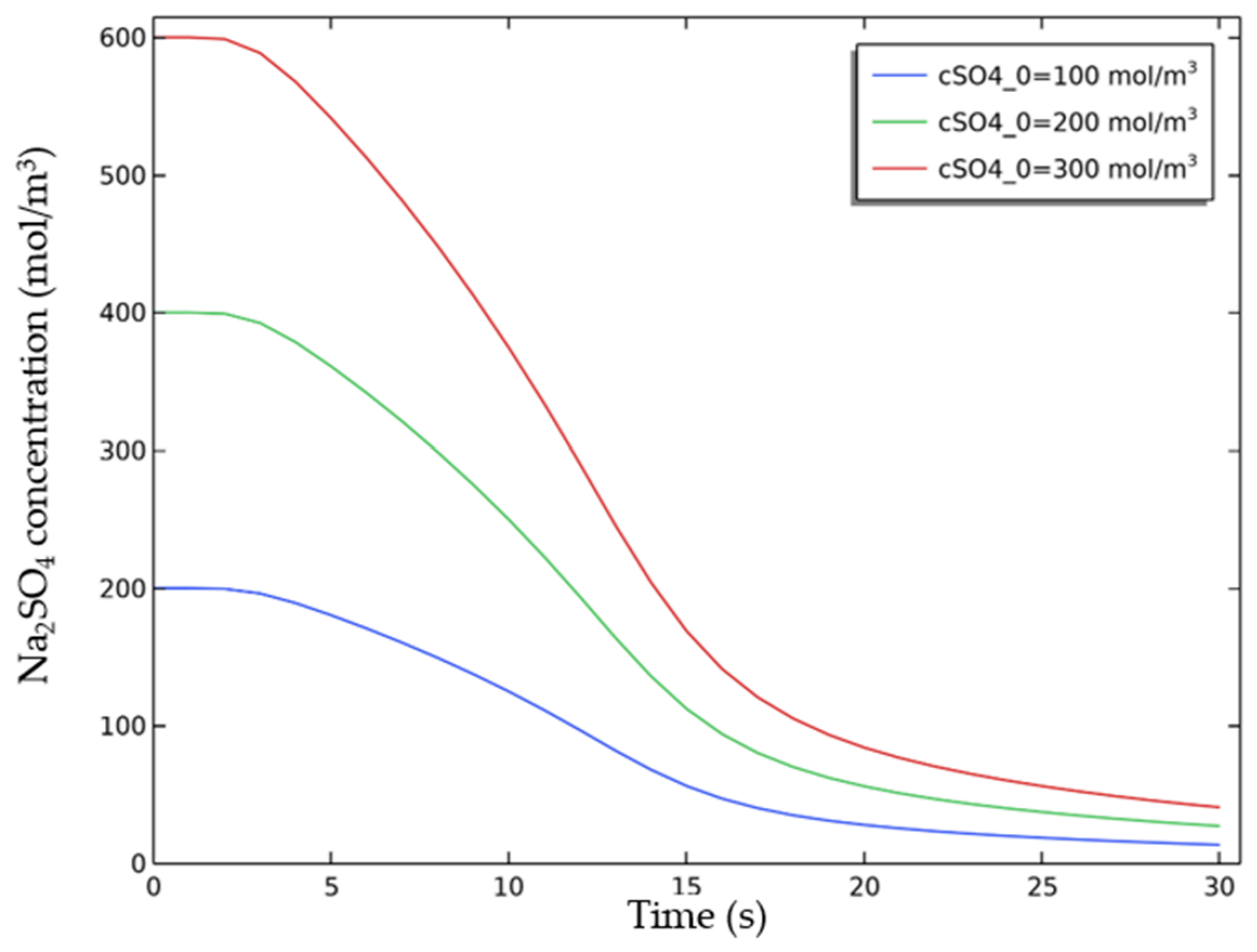
Simulated effect of initial concentration on the desalination profile.

Figure 10 shows the effect of the initial feed concentration on ED performance. Because the initial salt loads differed among the experimental groups, the desalination curves alone could not be used to compare the intrinsic desalination rates directly. Transient simulations were therefore performed (Figure 11). The simulation results indicated that the desalination rate increased with initial concentration. As with the voltage simulations, the transient desalination profiles shown in Figure 11 are intended for qualitative interpretation of the concentration effect trends and have not been quantitatively validated against the present experimental data. Consequently, although the initial Na_2_SO_4_ concentration was doubled from 34 to 68 g/L, the time required to reach the experimental endpoint increased by only approximately 15 min rather than doubling. Comparison of Figure 5a, Figure 8a and Figure 10a show that desalination rate remained relatively stable at high salinity but decreased at low salt concentrations.

**Figure 12:**
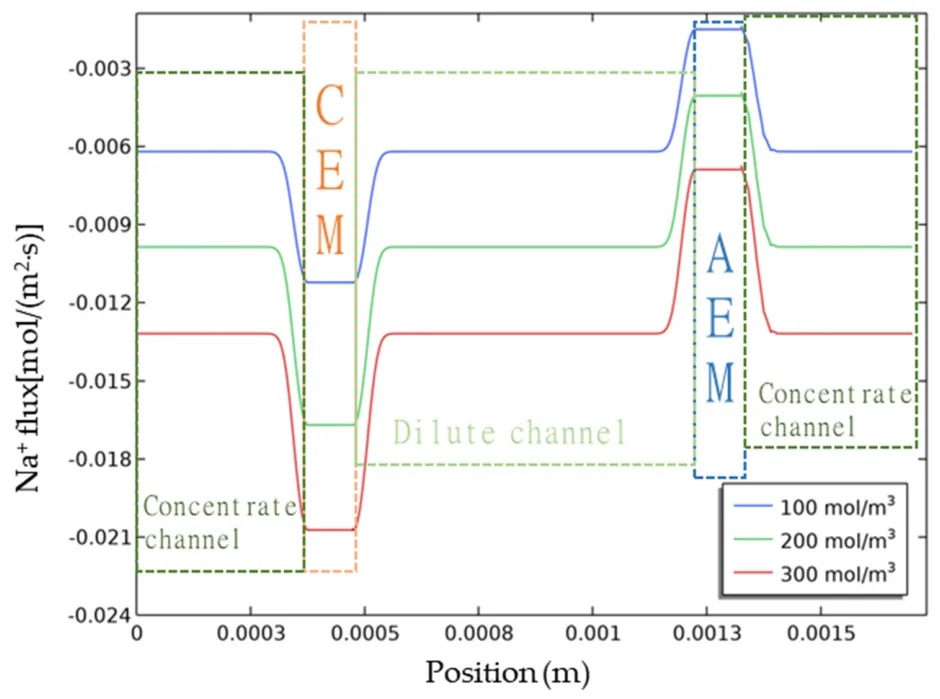
Simulated effect of initial concentration on the Na^+^ flux.

As shown in Figure 10b, the taurine recovery varied non-monotonically with feed concentration. Increasing the feed concentration exerted competing effects on the separation. a higher concentration increased the mass-transfer driving force and ion flux and reduced the solution resistance, resulting in a higher average current and a faster desalination rate (Figure 12). On the other hand, at high concentrations, the increased osmotic pressure and weaker Donnan exclusion may reduce membrane permselectivity [33,34], enhance water transport, and increase taurine loss, thereby decreasing product purity and recovery. Because the taurine/Na_2_SO_4_ mass ratio was held constant, both solute concentrations changed simultaneously, which may have contributed to the rebound in recovery at the highest concentration. Additional experiments in which the taurine concentration is held constant while only the Na_2_SO_4_ concentration is varied are required to isolate the effect of salt concentration on taurine loss.

Within the investigated range, an initial Na_2_SO_4_ concentration of 68 g/L provided a strong mass-transfer driving force without a pronounced loss of selectivity. Considering the desalination rate, product quality, and membrane productivity (Figure 10c), 68 g/L Na_2_SO_4_ was selected as the feed concentration for the subsequent experiments.

### 3.4. Separation Endpoint and Process-Control Analysis

**Figure 13:**
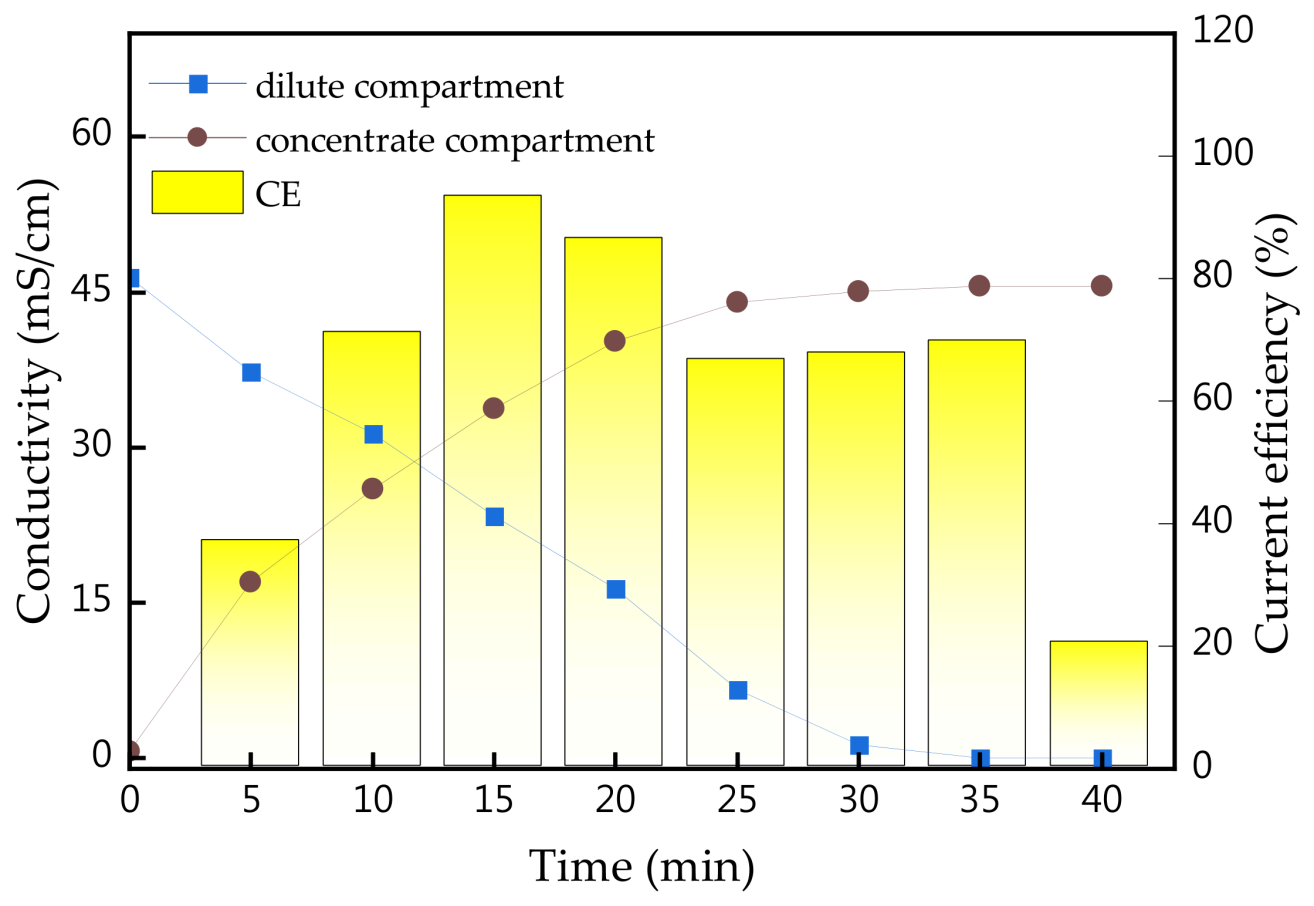
Changes in current efficiency and compartment conductivity during electrodialysis.

At 14 V, a circulation flow rate of 200 L/h (corresponding to a membrane-surface linear velocity of 6.2 cm/s), and initial concentrations of 68 g/L Na_2_SO_4_ and 100 g/L taurine, the taurine purity and recovery were 99.8% and 98.9%, respectively. The specific electrical energy consumption was 2022.3 kJ/kg Na_2_SO_4_ (0.56 kWh/kg Na_2_SO_4_) and 0.49 kg Na_2_SO_4_/m^2^·h. Additional experiments were conducted under the optimized operating conditions. Figure 13 shows the evolution of current efficiency and the conductivities of the dilute and concentrate compartments during ED.

The current efficiency was substantially lower at the beginning and end of the batch than during the middle stage. The maximum current efficiency, 93.5% during 10–15 min, was observed when the conductivities of the two compartments were similar. The low initial current efficiency was attributed primarily to the high electrical resistance of the deionized water initially present in the concentrate compartment. For continuous operation, the salt concentration in the concentrate compartment should be maintained at a level corresponding to a conductivity above 15 mS/cm to avoid excessive resistance and low current efficiency.

A decrease in current efficiency near the end of batch operation is difficult to avoid completely. Because the final product is crystalline taurine, the subsequent crystallization step can be operated so that trace Na_2_SO_4_ remains in the mother liquor rather than co-crystallizing with taurine. Terminating ED before the desalination efficiency decreases sharply can therefore improve equipment utilization and reduce energy consumption. On the basis of the dilute-conductivity curve and current-efficiency profile in Figure 13, 30 min was selected as the preferred endpoint for this system. At this time, the dilute conductivity was 1.27 mS/cm, corresponding to an Na_2_SO_4_ concentration of 1.93 g/L. Setting the endpoint at approximately 2 g/L Na_2_SO_4_ reduced the ED energy consumption by 16.5% relative to continued operation to the original endpoint.

To implement this endpoint-control strategy, the subsequent evaporation–crystallization step should be adjusted so that the solution is not evaporated to complete dryness. Instead, a small amount of water should be retained to keep part of the Na_2_SO_4_ in the liquid phase, and the concentrated mother liquor can then be recycled to the ED step. This strategy may support high taurine purity and recovery while enabling closed-loop mother-liquor circulation. Its feasibility was demonstrated only for the binary simulated taurine/Na_2_SO_4_ system. The present study was conducted with a binary simulated system. Industrial taurine mother liquor typically contains additional impurities such as sodium isethionate, sodium iminodisulfonate, ethylene glycol, and polyethylene glycol [33]. These impurities may affect the ED process through several mechanisms. First, studies on amino acid electrodialysis have shown that prolonged contact between ion-exchange membranes and organic solutes can lead to structural changes in the membrane material and irreversible performance decay [17]. Second, the presence of additional ionic species (sodium iminodisulfonate) may compete with Na^+^ and SO_4_^2−^ for transport through the membranes, potentially reducing the selectivity for salt removal and increasing taurine loss. Third, the accumulation of non-volatile impurities in the recycled mother liquor during closed-loop operation may require additional purification steps to maintain process stability. Therefore, before industrial implementation, the long-term stability of membrane performance, effective cleaning protocols, and impurity accumulation management must be further investigated using real mother liquor or impurity-spiked simulated solutions [35].

### 3.5. Comparison with Conventional Crystallization

The selected ED process followed by evaporation drying was compared with a conventional multistage crystallization process on the basis of an annual taurine production capacity of 500 t [9]. The raw synthesis mother liquor contained taurine and Na_2_SO_4_ at a mass ratio of 1:0.68. For the crystallization route, the recycled mother liquor contained 20% of the initial taurine mass, whereas the ED feed was diluted to a taurine mass fraction of 8.6% on the basis of the selected operating conditions. The results calculated per kilogram of taurine product are summarized in Table 5. A crystallization data from an industrial process package and calculated by the authors. ED total energy = ED electrical energy (0.56 kWh/kg) + evaporation energy (7.02 kWh/kg). Evaporation energy was estimated from water latent heat assuming 100% thermal efficiency. Auxiliary energies are not included. Within the defined accounting boundary, the ED route provided a higher taurine recovery and lower estimated energy consumption and separation cost.

As shown in Table 5, most of the energy consumption of the ED route arose from the subsequent evaporation step (7.02 kWh/kg taurine). The product obtained by direct evaporation drying after ED had a slightly lower purity than the recrystallized product from the conventional route and could be subjected to an additional polishing crystallization step if required. Within the stated accounting boundary, which excluded labor, maintenance, cleaning, and waste-liquor treatment, the ED route increased taurine recovery by 7.4 percentage points, reduced the specific energy consumption by 2.7 kWh/kg taurine, and lowered the estimated separation cost by 0.08 USD/kg taurine compared with conventional crystallization. However, this comparison is simplified and does not include auxiliary energy or long-term membrane replacement costs.

To assess the robustness of the economic evaluation, a simple sensitivity analysis was performed on the key assumptions listed above (Table 6). Each parameter was varied individually within a reasonable range while keeping all others at their baseline values, and the resulting change in the estimated separation cost was recorded.

As summarized in Table 6, the separation cost is most sensitive to steam price and membrane lifetime: increasing the steam price increases the cost by 25%, while reducing the membrane lifetime from 5 to 3 years increases the cost by 8.3%. In contrast, the ED separation cost is relatively insensitive to electricity price membrane price and annual operating hours have a moderate impact (+2.1%). Within the selected variation ranges, the ED separation cost remained consistently lower than that of conventional crystallization.

## 4. Conclusions

The laboratory-scale ED process was evaluated for separating taurine from Na_2_SO_4_ in a binary simulated mother liquor. Under the selected conditions (an applied voltage of 14 V, a circulation flow rate of 200 L/h, an initial Na_2_SO_4_ concentration of 68 g/L, and an initial taurine concentration of 100 g/L), the process achieved a taurine purity of 99.8% and a recovery of 98.9%. Compared with the conventional route within the defined accounting boundary (Table 5), the calculated taurine recovery increased by 7.4 percentage points.

Beyond process optimization, this study provides a practical strategy for process control: using approximately 2 g/L Na_2_SO_4_ in the dilute compartment as the separation endpoint. Unlike most previous ED studies that focus primarily on parametric optimization, we have quantitatively demonstrated that the low-salt stage is the dominant factor governing process energy efficiency. Setting the endpoint at 2 g/L Na_2_SO_4_ avoided inefficient operation at low salt concentrations and reduced the ED-unit energy consumption by approximately 16.5%. This endpoint-control strategy is simple, requires no additional equipment, and can be readily implemented in industrial practice. The estimated specific energy consumption and separation cost were lower by 2.7 kWh/kg taurine and 0.08 USD/kg taurine, respectively, than those of the conventional crystallization route. Coupling ED with controlled evaporation-crystallization may also reduce the direct discharge of salt-containing mother liquor. If higher product purity is required, a polishing crystallization step can be incorporated.

These results demonstrate the feasibility of electrodialytic desalination for a binary simulated taurine/Na_2_SO_4_ stream and indicate that an ED-crystallization process warrants further evaluation using real mother liquor and long-term continuous operation. In industrial taurine mother liquor, organic impurities and inorganic scaling may cause membrane fouling. Although various cleaning strategies exist, complete restoration of membrane performance is often difficult. Membrane lifetime under such conditions remains unknown and requires long-term pilot studies. Finally, it is acknowledged that the COMSOL model presented in this study has not been quantitatively validated against experimental data. The model is intended as a qualitative tool to support the interpretation of experimental trends rather than as a predictive simulation. Future studies with more comprehensive model parameters and systematic experimental validation could extend the quantitative capabilities of the modeling approach.

## Figures and Tables

**Table 1:** Specifications of the SED-30 electrodialysis system.

Parameters	Value
Cell pairs	10
Spacer thickness (mm)	0.75
Spacer size (cm^2^)	16 × 21
Membrane thickness (mm)	0.10–0.12
The commercial name of AEM	AC
The commercial name of CEM	CC
Membrane resistance of AEM (Ω∙cm^2^)	4.0–5.0
Membrane resistance of CEM (Ω∙cm^2^)	2.0–3.0
Effective membrane size (cm^2^)	12 × 17.5
Permselectivity (%)	96
Operating temperature range (°C)	<40
Operating pH range	1–9
Rectifier Output	30 V DC × 10 A

**Table 2:** Parameters used in the electrodialysis model.

Parameters	Value
Na^+^ diffusion coefficient (m^2^/s)	1.33 × 10^−9^
SO_4_^2−^ diffusion coefficient (m^2^/s)	1.06 × 10^−9^
Temperature (K)	298
Domain Height (m)	0.1
Membrane thickness (mm)	0.1
Compartment width (mm)	0.75
membrane ion concentration (mol/m^3^)	1000

**Table 3:** Solution temperature before and after electrodialysis at different applied voltages (mean ± SD, n = 3).

Voltage (V)	Initial Temperature (°C)	Final Temperature (°C)	Temperature Increase (°C)
8	24.8 ± 0.2	25.1 ± 0.2	0.3 ± 0.1
10	23.5 ± 0.0	23.8 ± 0.2	0.3 ± 0.1
12	22.1 ± 0.1	22.6 ± 0.3	0.5 ± 0.2
14	24.1 ± 0.2	25.1 ± 0.2	1.0 ± 0.2
16	24.6 ± 0.1	26.0 ± 0.3	1.4 ± 0.3

**Table 4:** Effect of applied voltage on the estimated electrodialysis separation cost.

Voltage (V)	Electricity Cost (USD/kg Taurine)	Membrane Cost (USD/kg Taurine)	Total Cost (USD/kg Taurine)
8	0.021	0.086	0.107
10	0.030	0.049	0.079
12	0.036	0.034	0.070
14	0.041	0.027	0.068
16	0.050	0.030	0.080

**Table 5:** Comparison of the conventional crystallization and electrodialysis routes. Mass- and cost-based quantities are reported per kilogram of taurine.

Parameter	Crystallization	Electrodialysis
Taurine purity (%)	100	99.8
Taurine recovery (%)	91.3	98.9
Waste generation (kg/kg taurine)	1.08	0 ^1^
Specific energy consumption (kJ/kg taurine)	10.1	7.4
Electricity cost (USD/kg taurine)	0.12	0.04
Steam cost (USD/kg taurine)	0.44	0.41
Membrane cost (USD/kg taurine)	-	0.03
Estimated separation cost (USD/kg taurine)	0.56	0.48

^1^ “0” waste refers to no direct discharge of salt-containing mother liquor under the defined closed-loop assumption and does not imply zero liquid discharge.

**Table 6:** Sensitivity analysis of key economic parameters on the estimated ED separation cost.

Parameter	Variation	Crystallization Cost (USD/kg Taurine)	Change from Baseline	ED Cost (USD/kg Taurine)	Change from Baseline
Baseline	-	0.56	-	0.48	-
Electricity price	+30%	0.60	7.1%	0.49	2.1%
Steam price	+30%	0.69	17.9%	0.60	25%
Membrane lifetime	3 year	0.56	-	0.52	8.3%
Membrane price	+30%	0.56	-	0.49	2.1%
Annual operating hours	6000 h	0.56	-	0.49	2.1%

## Data Availability

The data supporting the findings of this study are included in the article. Additional numerical data are available from the corresponding author upon reasonable request.
